# P-1410. Exposure to Potential Environmental and Zoonotic Reservoirs of Mycobacterium leprae Among Individuals with Hansen’s disease, Central Florida

**DOI:** 10.1093/ofid/ofaf695.1597

**Published:** 2026-01-11

**Authors:** Shamika V Chavda, Charlotte Avanzi, Tolulope Ojo-Akosile, DaJhe Sullivan, Annemieke Geluk, Paul L A M Corstjens, Norman Beatty, Kartikeya Cherabuddi, Andrew Miner, Jessica K Fairley

**Affiliations:** Emory University, Atlanta, GA; Colorado State University, Fort Collins, Colorado; Emory University School of Medicine, Atlanta, Georgia; Brevard Skin and Cancer Center, Rockledge, Florida; University of Leiden, Leiden, Zuid-Holland, Netherlands; University of Leiden, Leiden, Zuid-Holland, Netherlands; University of Florida, Gainesville, Florida; University of South Florida - Tampa General Hospital, Tampa, Florida; Brevard Skin and Cancer Center, Rockledge, Florida; Emory University, Division of Infectious Diseases, Atlanta, Georgia

## Abstract

**Background:**

Hansen’s disease (HD, leprosy), caused by *Mycobacterium leprae and M. lepromatosis*, remains endemic in parts of the southeastern United States with increasing incidence in Florida. In 2023, Florida accounted for 23 of 225 U.S. cases. The nine-banded armadillo is a known reservoir for *M. leprae* with mounting evidence of its role in transmission. *M. leprae* has also shown viability in soil, prompting examination of environmental exposures. Therefore, we aimed to explore environmental exposures in those with HD versus household contacts

**Table 1.**
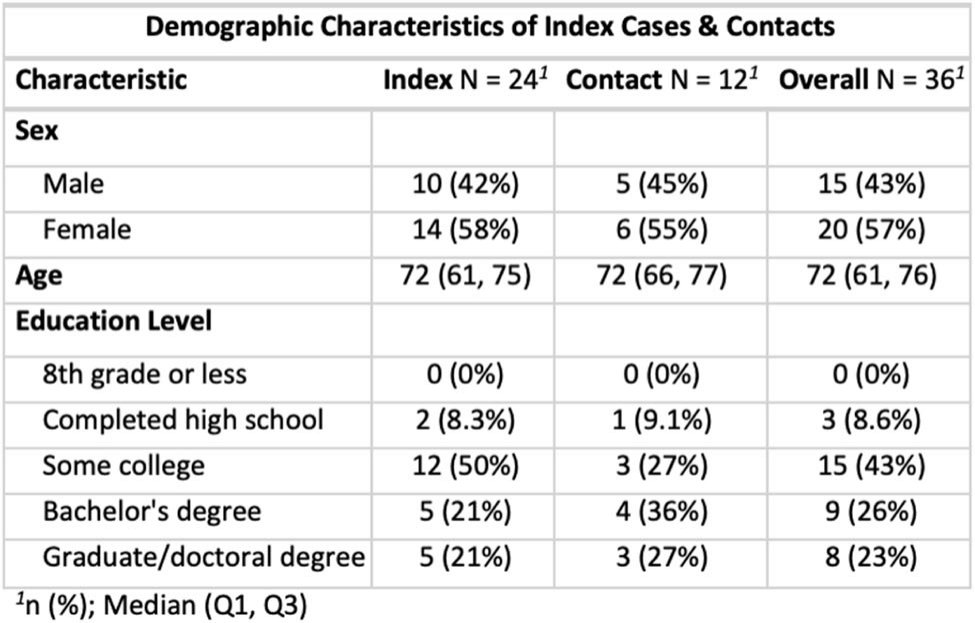
Demographic Characteristics of Index Cases & Contacts

**Table 2.**
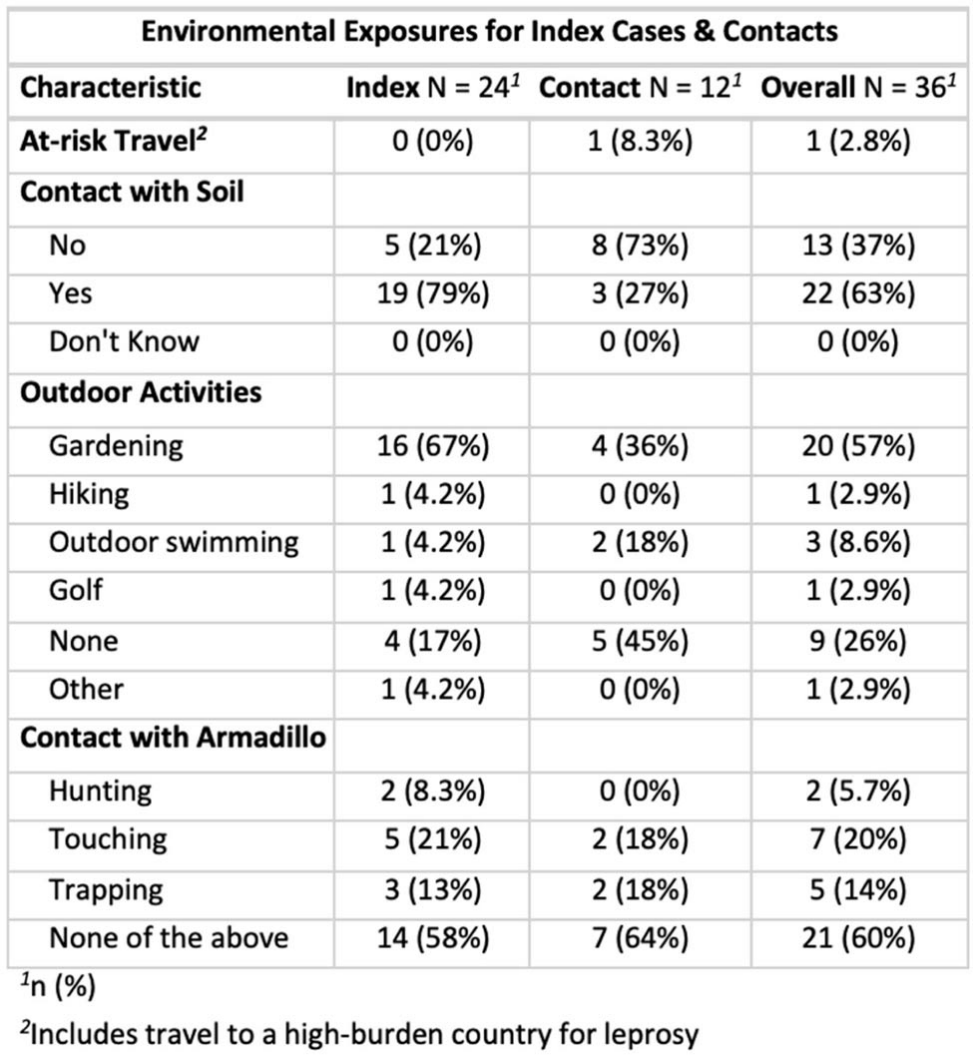
Environmental Exposures for Index Cases & Contacts

**Methods:**

We recruited participants from private dermatology clinics in East/Central Florida. Eligible individuals included those diagnosed with HD in the past 10 years and household contacts who lived with them for at least 6 months prior to diagnosis and treatment. Structured questionnaires were administered to collect data on age, sex, residence history, travel, and environmental exposure, including soil and armadillo contact.

**Results:**

We enrolled 36 individuals: 24 with HD and 12 household contacts without HD. Participants had a median age of 72 (53, 92); 20 (55%) were female. All individuals were U.S. born or were residents for 50+ years. 19 (79%) index cases and 3 (27%). Contacts reported regular soil contact (p=0.003). 16 (67%) index cases and 4 (36%) contacts reported gardening (p=0.07). 5 (21%) index cases and 2 (18%) contacts reported touching armadillos (p=0.6), 3 (13%) index cases and 2 (18%) contacts reported trapping them (p=0.5), and 2 (8.3%) index cases reported hunting them (p=0.4). 1 contact (8.3%) reported travel to a high-risk country for HD (p=0.3).

**Conclusion:**

This study showed frequent exposure to soil in those with HD that statistically differed from contacts. Typical risk factors like travel to, foreign residence in, or immigration from risk areas were absent. Armadillo contact did not differ greatly between cases and contacts, suggesting that the environmental route may be important in the epidemiologic triangle between humans and armadillos. Considering the ongoing detection of HD in Florida, particularly in East/Central Florida, future studies should continue to elucidate the role of contact with animal and environmental exposures in transmission.

**Disclosures:**

All Authors: No reported disclosures

